# Trajectories of Sense of Purpose Later in Life: A Coordinated Analysis of Six Longitudinal Studies

**DOI:** 10.1093/geroni/igaf122.3676

**Published:** 2025-12-31

**Authors:** Olivia Duchow, Gabrielle Pfund

**Affiliations:** Auburn University, Davis, California, United States; Auburn University, Auburn, Alabama, United States

## Abstract

A sense of purpose is defined as the extent to which individuals perceive their lives as having direction, goals, and meaning. Additionally, having a higher sense of purpose is consistently linked to healthier aging outcomes in older adulthood, including slower cognitive decline, reduced mortality risk, and less allostatic load. However, despite its importance, the trajectory of purpose across the lifespan remains understudied, particularly in later life. In this pre-registered work, we examined age-related trajectories of purpose using coordinated data analysis across six longitudinal studies: Health and Retirement Study (N = 14,000), Midlife in the United States (N = 4,000), Rush Memory and Aging Project (N = 2,300), Rush Minority Aging Research Study (N = 950), Rush Latino Core (N = 250), and Wisconsin Longitudinal Study (N = 10,300). These datasets include diverse populations with at least three measurement occasions of purpose with individuals spanning years to decades. These strengths enable the harmonized examination of purpose development within adults over time. We incorporated demographic and health covariates including race, gender, education, relationship status, and health conditions to examine their impact on purpose trajectories. Using a coordinated analysis approach, this study used multilevel linear and quadratic growth models to characterize how sense of purpose changes as individuals age across diverse groups. Sense of purpose tends to decline later in the lifespan, highlighting the vulnerability of older adults to maintain a sense of purpose. Findings inform the importance of future intervention development aiming to promote purpose into older adulthood.

